# Supplementary figures and images for: Interactions between genetic variation and cellular environment in skeletal muscle gene expression
Source: PLoS One. 2018 Apr 16;13(4):e0195788. doi: 10.1371/journal.pone.0195788 (PMC5901994; doi:10.1371/journal.pone.0195788)

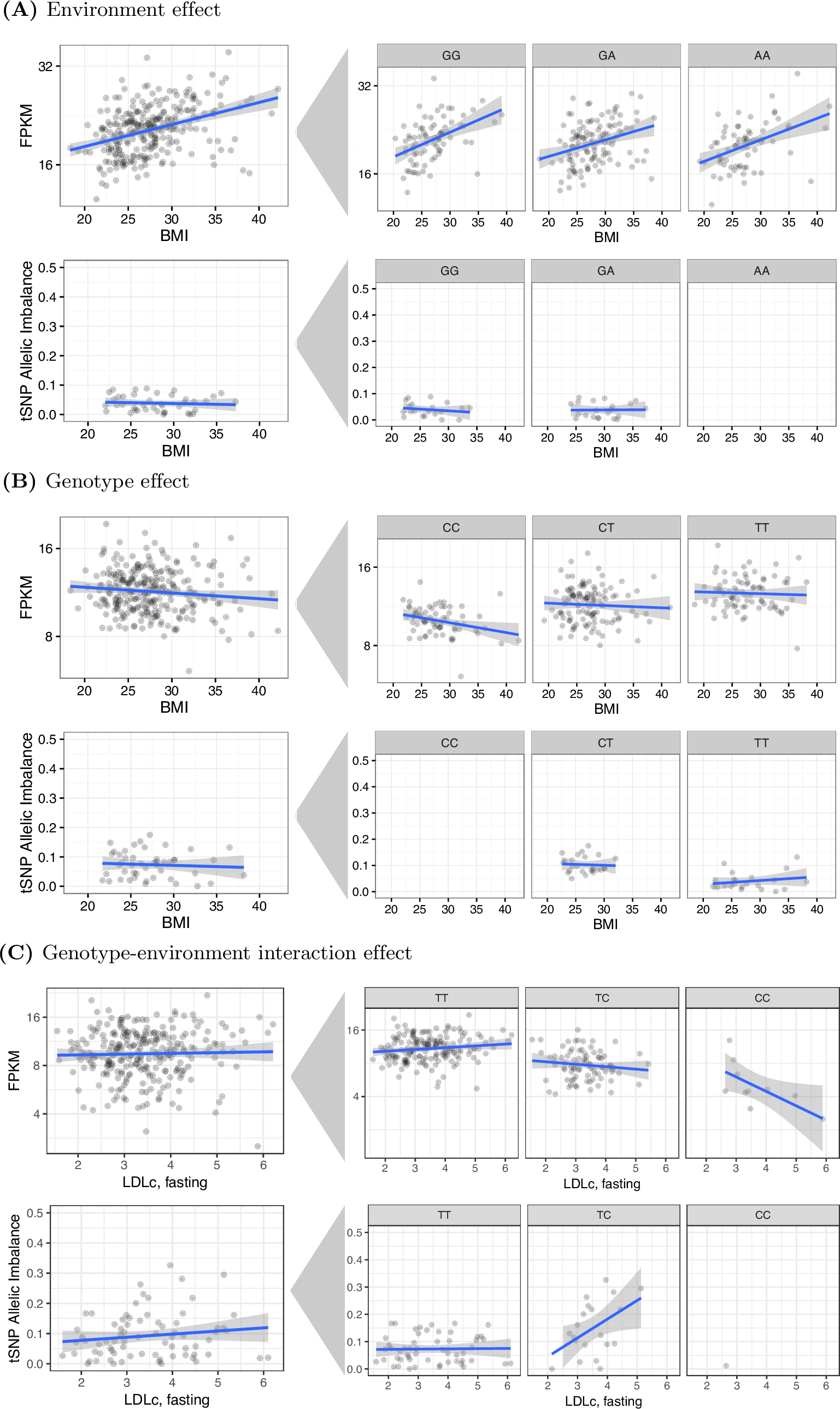

Supplement: S1 Fig — (A) Example of a pure environment effect in SZRD1—rs12568938 regulatory SNP (rSNP) and rs7529767 transcribed SNP (tSNP). SZRD1 expression is associated with BMI, and the rSNP does not affect gene expression. The relationship between SZRD1 and BMI does not change across the rSNP alleles, and BMI is not associated with allelic imbalance. (B) Example of a pure genetic effect in RBM6—rs9881008 regulatory locus and rs2023953 tSNP. BMI is not associated with RBM6 expression or allelic imbalance. The rSNP alleles are associated with RBM6 expression and allelic imbalance is increased in samples heterozygous for the rSNP. (C) Example of a GxE effect in FHOD3—rs17746240 regulatory locus and rs72895597 tSNP. The relationship between LDLc and FHOD3 expression changes according to the rSNP allele as well as the overall expression abundance levels. LDLc is only associated with allelic imbalance in heterozygous individuals, where preferential TF binding could occur. (TIF) [file pone.0195788.s003.tif]

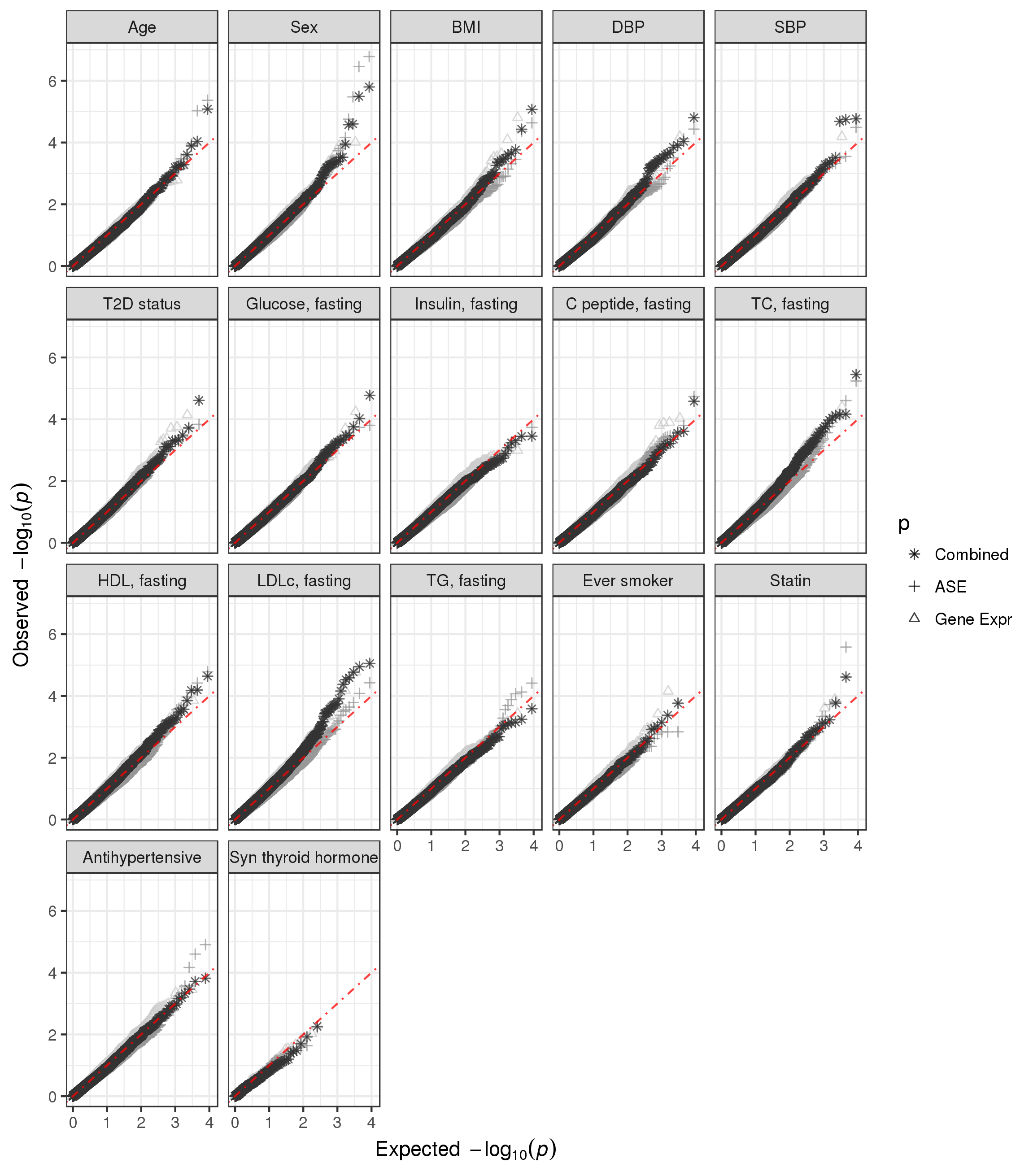

Supplement: S2 Fig — QQ-plots of GxE signal discovery across clinical traits. Colors and shapes depict the ASE, gene-level, and combined p-values. (TIF) [file pone.0195788.s004.tif]

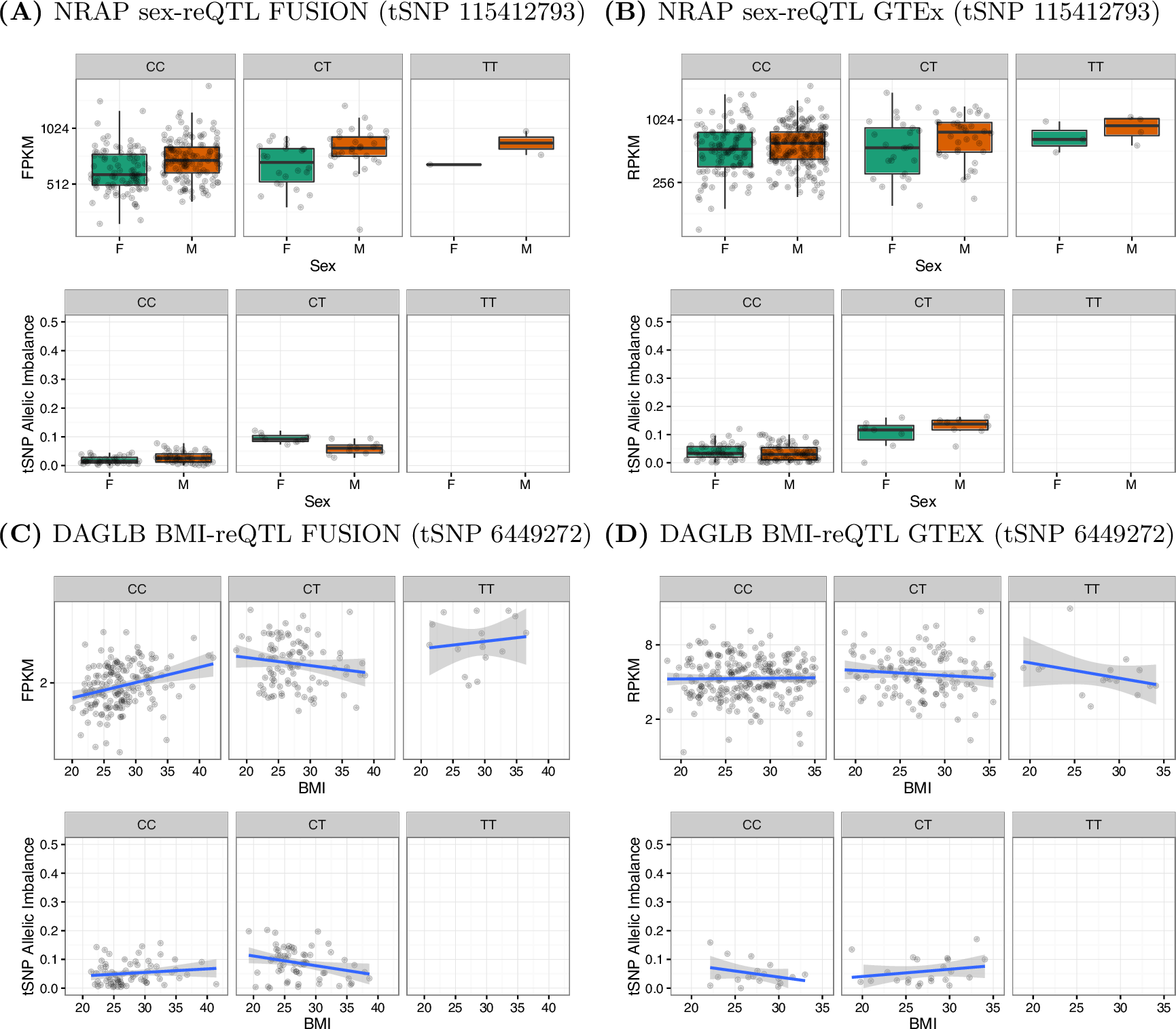

Supplement: S3 Fig — (A) NRAP sex-reQTL in FUSION. (B) NRAP sex-reQTL in GTEx. (C) DAGLB BMI-reQTL in FUSION. (D) DAGLB BMI-reQTL in GTEx. (TIF) [file pone.0195788.s005.tif]

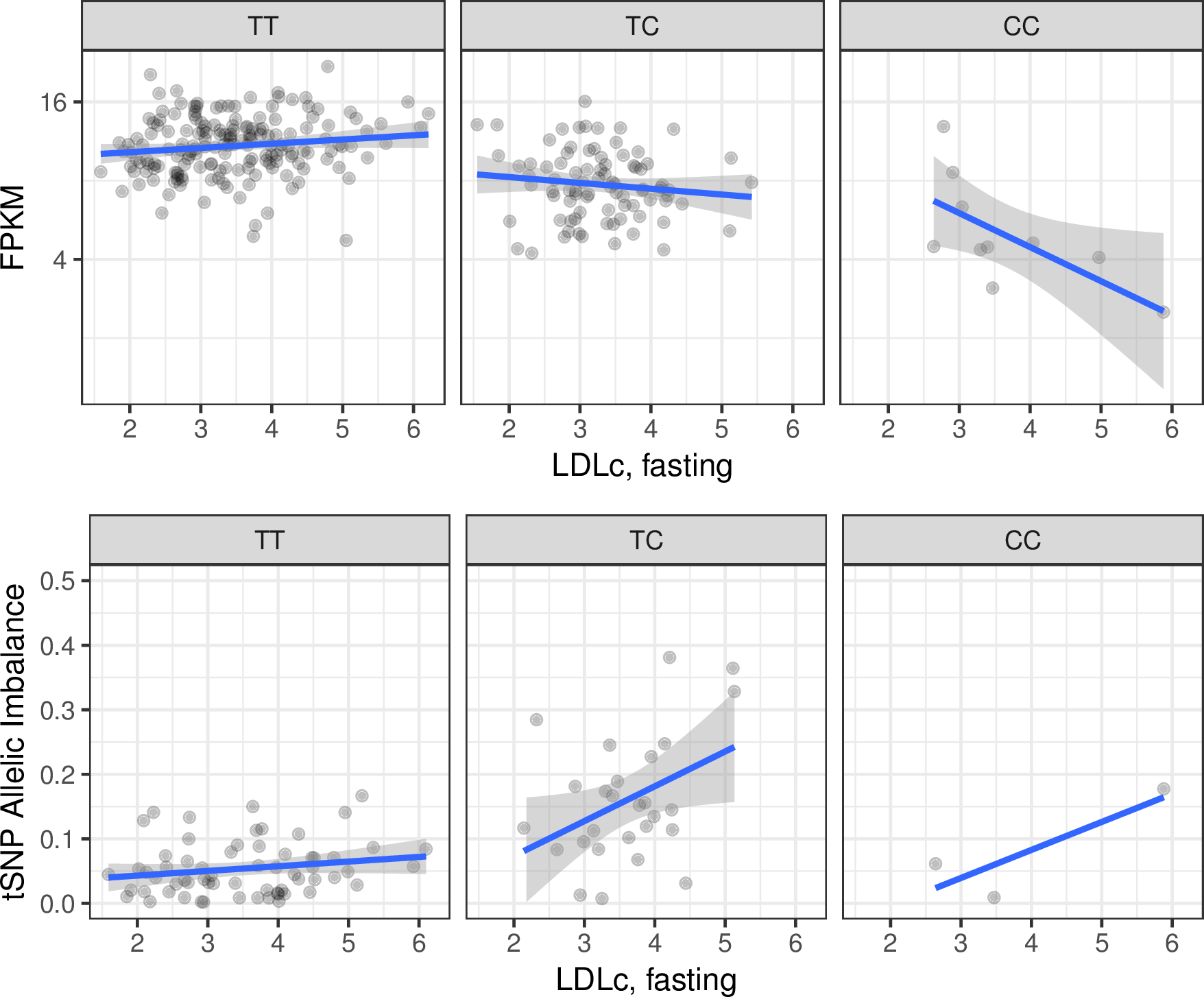

Supplement: S4 Fig — Additional LDLc GxE effect with rs61735993 (18:34273279) as the tSNP. (TIF) [file pone.0195788.s006.tif]

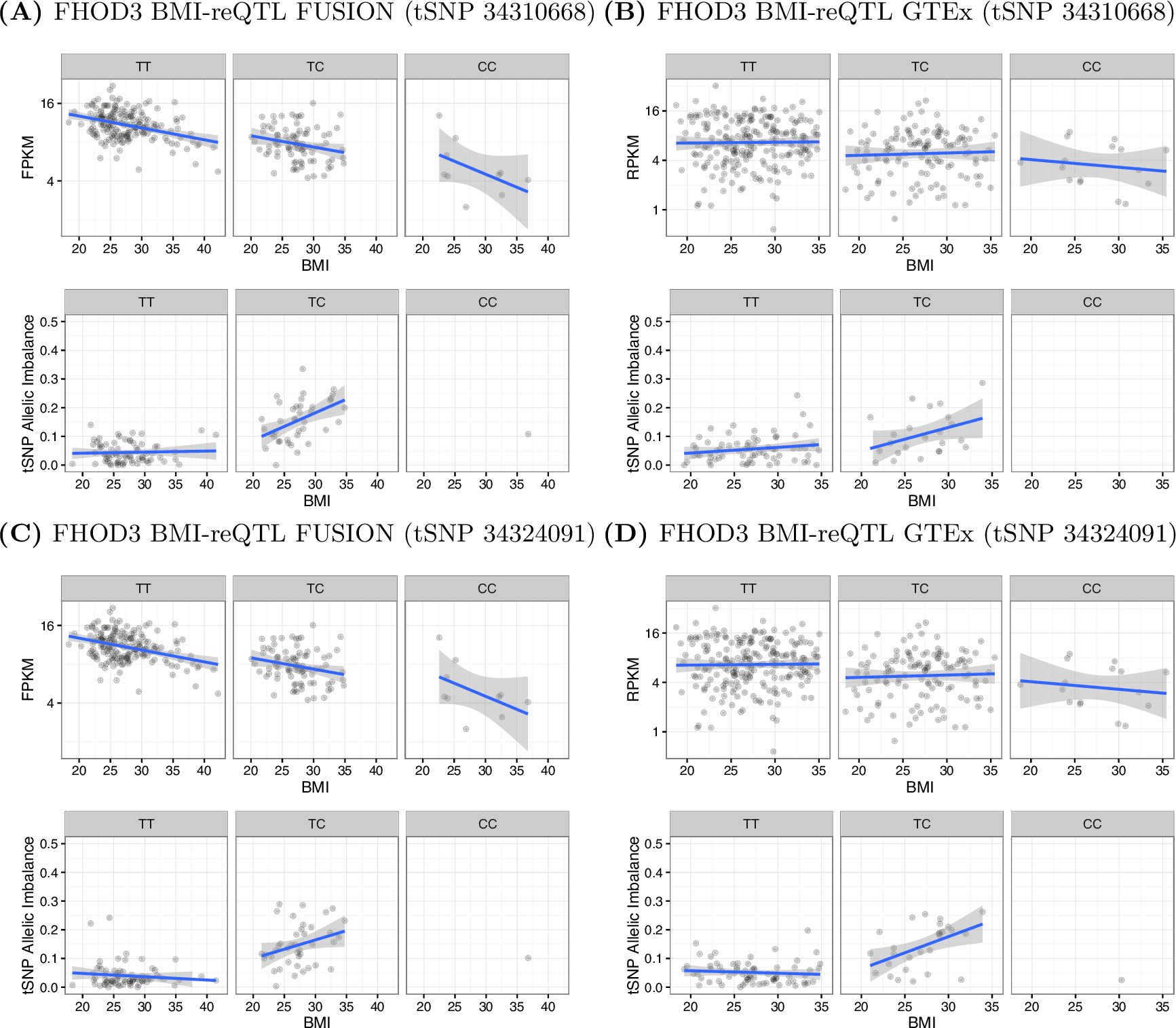

Supplement: S5 Fig — (A) FHOD3 BMI-reQTL in FUSION with rs3744903 (18:34310668) as the tSNP. (B) FHOD3 BMI-reQTL in GTEx with rs3744903 (18:34310668) as the tSNP. (C) FHOD3 BMI-reQTL in FUSION with rs2303510 (18:34324091) as the tSNP. (D) FHOD3 BMI-reQTL in GTEx with rs2303510 (18:34324091) as the tSNP. (TIF) [file pone.0195788.s007.tif]

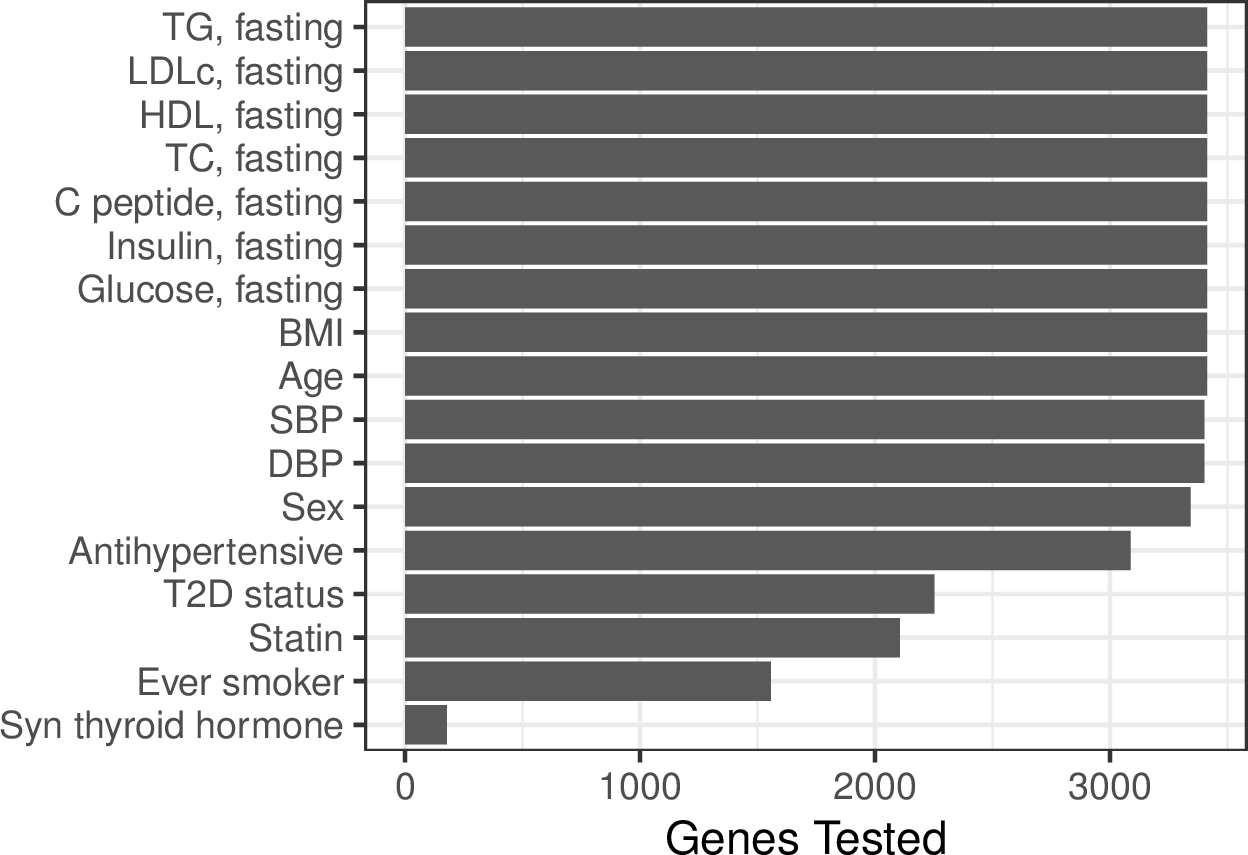

Supplement: S6 Fig — Total number of genes in FUSION considered for each clinical trait. (TIF) [file pone.0195788.s008.tif]

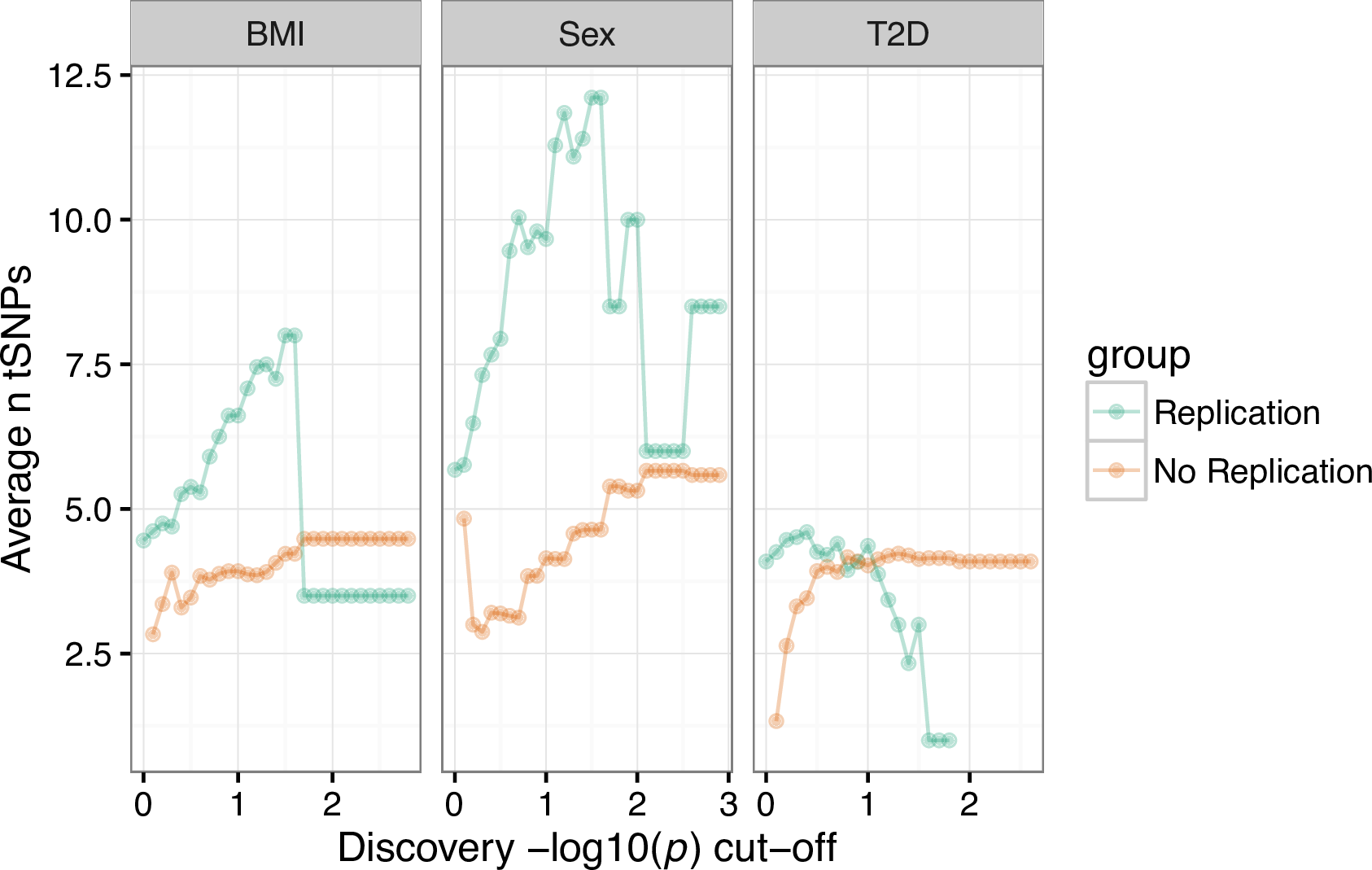

Supplement: S7 Fig — Average number of tSNPs in the genes with signals that replicated (Replication group) and signals that did not replicate (No Replication). (TIF) [file pone.0195788.s009.tif]
